# Real-time polymerase chain reaction (PCR) for *Pneumocystis jirovecii* detection in lower respiratory tract samples

**DOI:** 10.1128/spectrum.01563-25

**Published:** 2025-11-06

**Authors:** Federica Anna Maria Giardina, Marina Ramus, Francesca Campanini, Daniele Lilleri, Virginia Ferretti, Antonino Maria Guglielmo Pitrolo, Ambra Vola, Roberta Maserati, Fausto Baldanti, Stefania Paolucci

**Affiliations:** 1 Department of Clinical, Surgical, Diagnostic and Pediatric Sciences, University of Pavia 19001, Pavia, Italy; 2 UOC Microbiology and Virology, IRCCS Foundation Policlinico San Matteo 18631, Pavia, Italy; 3 Biostatistics and Clinical Trial Center, Fondazione IRCCS Policlinico San Matteo 18631, Pavia, Italy; University of Cincinnati, Cincinnati, Ohio, USA

**Keywords:** *Pneumocystis jirovecii*, qPCR, quantification, colonization, cut-off

## Abstract

To date, the gold standard for *Pneumocystis jirovecii* pneumonia (PjP) diagnosis is direct microscopic examination (ME). However, several studies have shown that the DNA copy number of the *P. jirovecii*, measured by quantitative real-time PCR (qPCR), is significantly higher in patients with PjP than in colonized individuals. Nevertheless, this approach is not standardized yet and requires further investigation. In this study, we aimed to compare the results obtained using the Pneumocystis ELITe MGB Kit (ELITech Group S.p.a., Italy) with those from ME, evaluating specificity, sensitivity, and the ability to distinguish infection from colonization through the identification of a cut-off value. A total of 163 bronchoalveolar lavage (BAL) or sputum samples, collected and tested with direct ME for *P. jirovecii* in our Unit over the past 10 years, were also analyzed using a qPCR assay. The results were concordant between the two methods in all 63 ME-positive samples. Among 100 ME-negative samples, 21 (21%) tested positive by qPCR. A receiver operating characteristic curve analysis was performed to assess the qPCR assay’s prognostic performance in identifying patients already diagnosed with PjP by ME on BAL or sputum samples. A cut-off value of 150 DNA copies/10^5^ cells yielded a sensitivity of 100% (95% confidence interval [CI]: 94%–100%) and a specificity of 99% (95% CI: 95%–100%) in the diagnosis of the disease. qPCR may be used alongside careful clinical evaluation to ensure accurate diagnosis and appropriate treatment.

**IMPORTANCE:**

Recently, molecular approaches have been proposed for the detection of *Pneumocystis jirovecii*. Thanks to their high sensitivity, these methods can detect small amounts of fungal DNA. However, it is necessary to establish a cut-off value to distinguish between colonization and infection. Since *P. jirovecii* is closely related to epithelial cells in the lung environment, normalizing the fungal DNA load on the host cellular DNA content could represent an important step toward standardizing the procedure.

## INTRODUCTION

*Pneumocystis jirovecii* is an opportunistic fungus belonging to the phylum Ascomycota, transmitted via the airborne route. It is the causative agent of *Pneumocystis jirovecii* pneumonia (PjP), also referred to as *Pneumocystis* pneumonia, which is characterized by interstitial pneumonia, fever, non-productive cough, and dyspnea.

PjP mainly affects immunocompromised individuals, such as solid organ transplant recipients or patients with hematological malignancies (1, 2). With the advent of HIV and AIDS, its prevalence increased dramatically, making it one of the most significant life-threatening infections in this population (3). However, cases of PjP had also been described in individuals without any proven immunodeficiency (4, 5).

Moreover, asymptomatic pulmonary colonization may occur in immunocompetent subjects, such as children, elderly individuals with lung diseases (6), and health care workers (7, 8) who may serve as reservoirs for the transmission of *P. jirovecii* to immunocompromised patients (9).

The gold standard for PjP diagnosis remains direct microscopic examination (ME) on lower respiratory tract samples after appropriate staining. However, due to its low sensitivity and inability to differentiate between colonization and active infection, there has been a progressive shift in the literature toward molecular diagnostic methods. Real-time PCR assays proved to be more sensitive and specific, increasing detection rates up to fivefold compared to ME (10, 11).

In recent years, several study groups have reported on the diagnostic performance of different molecular assays. In 2021, Liotti and colleagues developed a new in-house assay and highlighted the possibility of using a single-copy gene target rather than a multiple-copy one (12). Furthermore, due to the clinical relevance of invasive fungal diseases (IFDs) in immunocompromised patients, a multiplex real-time PCR assay for the simultaneous detection of *Aspergillus fumigatus* and *P. jirovecii* was developed (13).

The preferred sample for PjP diagnosis is bronchoalveolar lavage (BAL). Very recently, Ghelfenstein-Ferreira and colleagues evaluated the diagnostic performance of quantitative real-time PCR (qPCR) assays on different BAL fractions (neat BAL, pellet, and supernatant obtained by centrifugation). Their findings demonstrated that both the whole BAL and pellet fractions yielded comparable results in terms of sensitivity and quantification, whereas the supernatant produced lower positivity rates (14).

Sputum samples, by contrast, are not the elective sample for PjP diagnosis and can lead to difficulties in the identification process and false negatives in ME (15).

The role of colonization was not fully understood until the development of *Pneumocystis*-specific molecular assays (16), which enabled the detection of low levels of fungal DNA (17). Several studies have demonstrated that the DNA copy number of *P. jirovecii*, as measured by qPCR, is significantly higher in patients with PjP than in colonized individuals. Moreover, some authors have proposed using molecular assays to define threshold values capable of distinguishing colonization from infection. However, this approach has not yet been standardized and warrants further investigation (18).

Thus, molecular assays can complement traditional methods to increase sensitivity, particularly in patients for whom BAL sampling is not feasible and sputum is the only available specimen (19).

The aim of this monocentric, retrospective, observational, and cross-sectional study is to investigate the use of molecular biology assays in a diagnostic setting. Specifically, we aimed to compare a commercial qPCR Kit with the gold standard ME in terms of diagnostic accuracy and the ability to differentiate colonization from infection, establishing a cut-off value. In addition, considering that *P. jirovecii* is closely related to the epithelial cells, we also aimed to evaluate the normalization of fungal load based on the cellular DNA, quantified by β2-microglobulin measurement.

## MATERIALS AND METHODS

### Patients and samples

All samples collected from patients with suspected PjP (according to National Guidelines [20]) were included in the study. Table S1 reports the clinical criteria for suspected PjP.

One hundred sixty-three residual samples (144 BAL and 19 sputum samples), collected and subjected to direct ME for *P. jirovecii* in the Parasitology Laboratory of our institution over the past 10 years, were included.

Samples were collected from 163 patients admitted to several departments of our institution. In particular, 48 (29.5%) were admitted to the Infectious Diseases Department, 48 (29.5%) to the Pneumology Department, 18 (11%) to the Intensive Care Unit (ICU), 14 (8.6%) to the Hematology Department, 6 (3.7%) to the Rheumatology Department, 2 (1.2%) to the Cardiac Surgery Department, 2 (1.2%) to the Pediatric Oncohematology, 1 (0.6%) to the Surgery Department, 1 (0.6%) to the Internal Medicine Department, 1 (0.6%) to the Gynecology Department. Additionally, 22/163 (13.5%) patients were admitted to other hospitals.

Clinical data were retrieved for all patients included in the study, except for 22 patients (13.5%) admitted to other hospitals and 5 patients (3.1%) admitted to our hospital, for 136 clinical records available.

Table 1 reports the demographic and clinical characteristics of the study population, retrieved from the hospital’s dedicated electronic systems.

**TABLE 1:** Patients’ demographic and clinical characteristics

	All patients (*n* = 163)	PjP patients (*n* = 63)
*Characteristics*		
Median age (range)	56 (7–84)	52 (14–81)
Sex (%)		
Male	93 (57)	47 (75)
Female	70 (43)	16 (25)
*Risk factor (n [%])*		
HIV infection	33 (20.2)	23 (36.5)
Hematological malignancies	33 (20.2)	10 (15.8)
Lymphoma	6 (3.7)	10 (15.8)
Leukemia	27 (16.5)	9 (14.3)
Solid organ transplantation	16 (9.8)	2 (3.2)
Kidney transplantation	2 (1.2)	0 (0)
Lung transplantation	10 (6.1)	0 (0)
Heart transplantation	3 (1.8)	2 (3.2)
Liver transplantation	1 (0.6)	0 (0)
Solid malignancies	8 (4.9)	0 (0)
Autoimmune diseases with immunosuppressive regimens	20 (12.3)	0 (0)
Combined immunocompromised	8 (4.9)	4 (4.7)
HIV + autoimmune diseases	2 (1.2)	2 (3.2)
Lymphoma + solid malignancies	1 (0.6)	1 (1.6)
Autoimmune diseases + hematological malignancies	2 (0.6)	0 (0)
Autoimmune diseases + solid malignancies	3 (1.8)	1 (1.6)
Not defined	27 (16.5)	18 (28.5)
None/suspected	8 (4.9)	0 (0)

Clinical data allowed the assessment of the appropriateness of the pre-analytical request and the estimation of the degree of suspicion of infection. Moreover, the patients’ likelihood of developing the disease was classified as high probability (HP) and low probability (LP) according to well-known risk factors for PjP, including the presence or clinical suspect of immunocompromise, clinical or imaging records of interstitial pneumonia, and laboratory criteria (high level of lactate dehydrogenase, PCR, and procalcitonin).

Samples were subjected to ME after centrifugation and appropriate staining of the pellet with toluidine blue. As a standard procedure in our laboratory, residual respiratory samples were stored at −80°C after diagnostic procedures.

### β2-microglobulin quantification

To investigate if specimens were appropriately collected and then suitable for *Pneumocystis* detection and diagnosis, endogenous β2-microglobulin was quantified by qPCR assays (21).

Briefly, after appropriate nucleic acid extraction from 400 µL of samples with QIAsymphony DSP Virus/Pathogen Kit (Qiagen, Heidelberg, Germany), an amplification mix was prepared using QuantiFast Pathogen +IC Kit (Qiagen, Heidelberg, Germany), according to the manufacturer’s instructions. The thermal profile was as follows: 5 minutes at 95°C, followed by 45 cycles of 15 seconds at 95°C, and 30 seconds at 60°C. The amplification and quantification of β2-microglobulin were also used as an internal control to evaluate the proper extraction of nucleic acid.

### Molecular detection of *P. jirovecii*

*P. jirovecii* molecular detection was performed on the same DNA eluate, obtained for β2-microglobulin quantification, using Pneumocystis ELITe MGB Kit (ELITech Group S.p.a., Italy), according to the manufacturer’s instructions. Briefly, the ELITe MGB Kit is a CE-marked, IVDR-cleared quantitative assay for the amplification of the mitochondrial large Subunit of rRNA gene (mtLSU) of *P. jirovecii*, as also described by Jakab et al. (22).

Real-time assays were carried out using Rotor-Gene Q thermal cycler with the following thermal profile: 2 minutes at 50°C, 2 minutes at 94°C, followed by 45 cycles of 10 seconds at 94°C, 20 seconds at 60°C, and 20 seconds at 72°C. To obtain a reliable quantification of fungal DNA, four standard samples (PJ Q Standard 10^2^–10^5^) were tested in each assay. Fungal load was normalized based on β2-microglobulin so that the results were expressed as DNA copies/100,000 cells.

### Statistical analysis

Qualitative variables were described as percentages for each category. Quantitative variables were described in terms of median and interquartile range, depending on the distribution of the variable. If necessary, quantitative variables will be log-transformed. The Type I error rate will be set at 5%. All statistical analyses will be conducted using Stata 17 (StataCorp, USA, 2021).

The sensitivity of qPCR will be reported along with its 95% confidence interval (95% CI) estimated using the binomial method.

The inter-rater reliability between the different methods was calculated with Cohen’s Kappa (95% CI).

Receiver operating characteristic (ROC) curve analysis was performed to evaluate the optimal cut-offs to discriminate between patients with or without PjP. The cut-off was established according to Youden’s index (or Youden’s *J* statistic) (23), defined as follows: *J* = sensitivity + specificity − 1.

The area under the curve and its 95% CI were calculated. The analyses were performed using GraphPad Prism 9.1.0 (GraphPad Software Inc., La Jolla, CA, USA).

## RESULTS

This study aimed to investigate the use of molecular biology assays in a diagnostic setting.

Among 163 samples, 100 (61.3%) samples were negative and 63 (38.6%) were positive for *P. jirovecii* in ME.

**Fig 1:**
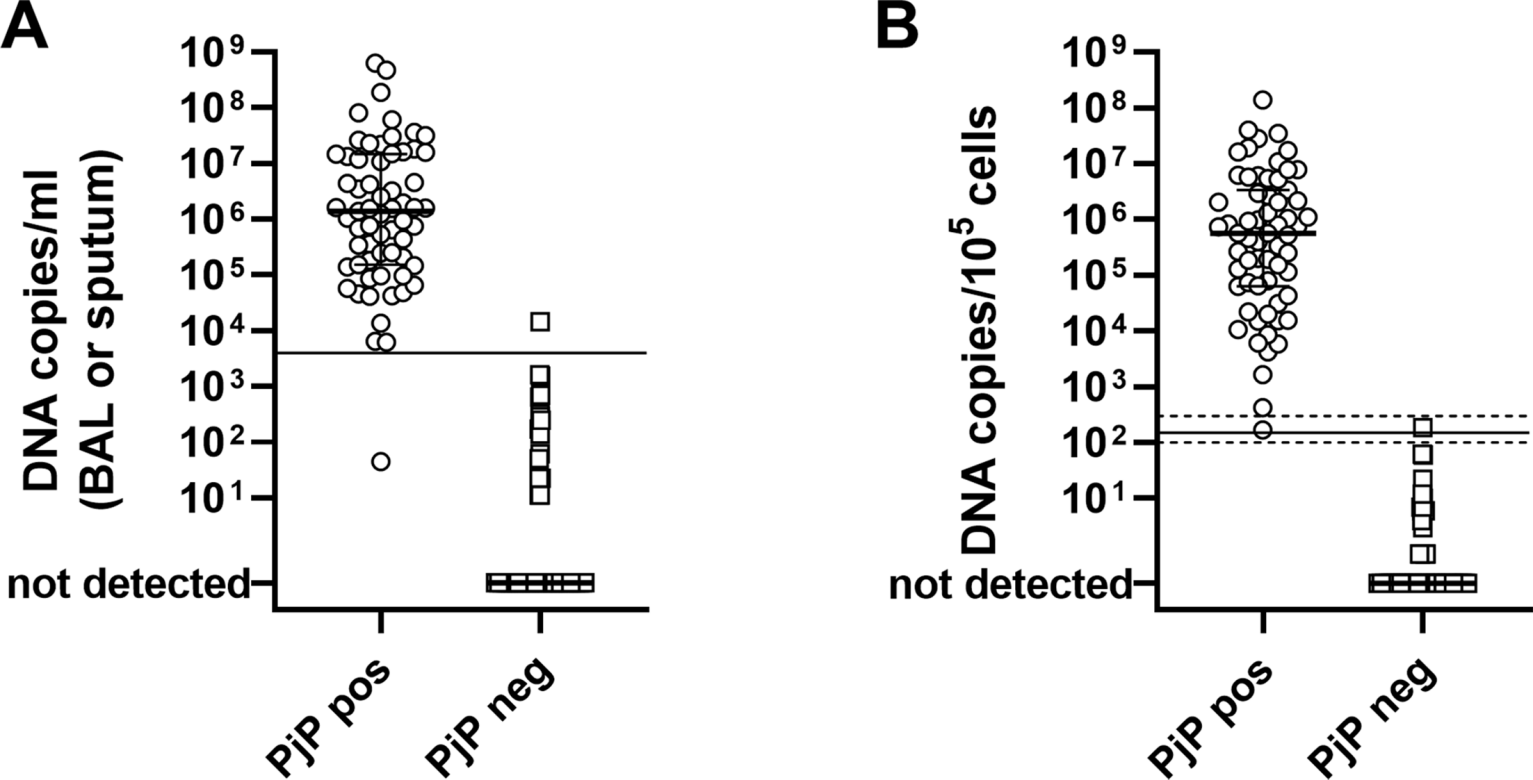
(**A**) *P. jirovecii* DNA load (copies/mL) in patients with positive (PjP pos) or negative (PjP neg) conventional diagnosis of PjP. (**B**) *P. jirovecii* DNA load normalized on cellular DNA content (copies/10^5^ cells). Horizontal lines represent the optimal cut-off value for diagnosis of PjP: 4,000 copies/mL (A) or 150 copies/10^5^ cells (B), whereas horizontal dashed lines represent boundaries of a borderline zone (100–300 copies/10^5^ cells).

All 63 ME-positive samples were also confirmed by qPCR, while 21/163 (12.9%) samples that were negative in ME resulted qPCR-positive. The concordance between ME and the qPCR method, calculated with Cohen’s kappa, was 0.77 (95% CI: 0.67–0.86). Among discordant samples, 20/21 (95.2%) had <2,000 *P*. *jirovecii* DNA copies/mL, whereas 1/21 (4.8%) had >14,000 *P*. *jirovecii* DNA copies/mL (Fig. 1A).

ROC curve analysis was performed to analyze the prognostic performance of the qPCR assay in the identification of patients with PjP as diagnosed by the conventional direct detection of the microorganism in BAL or sputum. A cut-off of 4,000 *P. jirovecii* DNA copies/mL BAL or sputum had 99% (95% CI: 95%–100%) specificity and 98% (95% CI: 92%–100%) sensitivity in the diagnosis of PjP (four patients with PjP had DNA load below the optimal cut-off). *P. jirovecii* DNA load values were then normalized on the cellular content of the samples, based on β2-microglobulin quantification (Fig. 1B). When reporting results on a standard number of 10^5^ cells examined, in 20/21 (95.2%) discordant samples, *P. jirovecii* load was lower than 100 DNA copies/10^5^ cells.

According to risk factors for PjP, including HIV infection, immunocompromise, clinical and radiological findings, flogosis, LDH levels, and PjP therapy, patients were classified into HP and LP groups.

Results of the classification are reported in Table 2.

**TABLE 2:** Clinical, biochemical, radiological findings, and outcomes in 163 patients

Infection probability	Immunocompromised		Clinical findings		Radiological findings		Flogosis		High LDH		Prophylaxis		Target therapy		Success	
	HIV	Non-HIV	Yes	No	Yes	No	Yes	No	Yes	No	Yes	No	Yes	No	Yes	No
HP with qPCR pos (%)	30 (18.4)	15 (9.2)	42 (25.8)	0 (0)	41 (25.1)	0 (0)	0 (0)	41 (25.1)	20 (12.3)	15 (9.2)	4 (2.4)	35 (21.5)	33 (20.2)	1 (0.6)	30 (18.4)	2 (1.2)
LP with qPCR neg (%)	2 (1.2)	57 (35.0)	47 (28.8)	8 (4.9)	17 (10.4)	38 (23.3)	21 (12.9)	24 (14.7)	9 (5.5)	21 (12.9)	3 (1.8)	51 (31.3)	6 (3.7)	24 (14.7)	28 (17.2)	1 (0.6)

The ROC curve analysis was then performed to analyze the prognostic performance of the qPCR assay results with reference to 10^5^ cells.

A cut-off value of 150 DNA copies/10^5^ cells had a sensitivity of 100% (95% CI: 94%–100%) and a specificity of 99% (95% CI: 95%–100%) in the diagnosis of the disease (Fig. 1B). All patients with PjP had DNA values above this cut-off, and only one patient without PjP had a DNA value slightly above the cut-off. Alternatively, we could define a borderline zone between 100 and 300 DNA copies/10^5^ cells: all the patients with <100 DNA copies/10^5^ cells did not have PjP, whereas all the patients with >300 DNA copies/10^5^ cells had PjP. Only 2/163 patients (1.2%) fell into the borderline zone: one with PjP and one without PjP.

**Fig 2:**
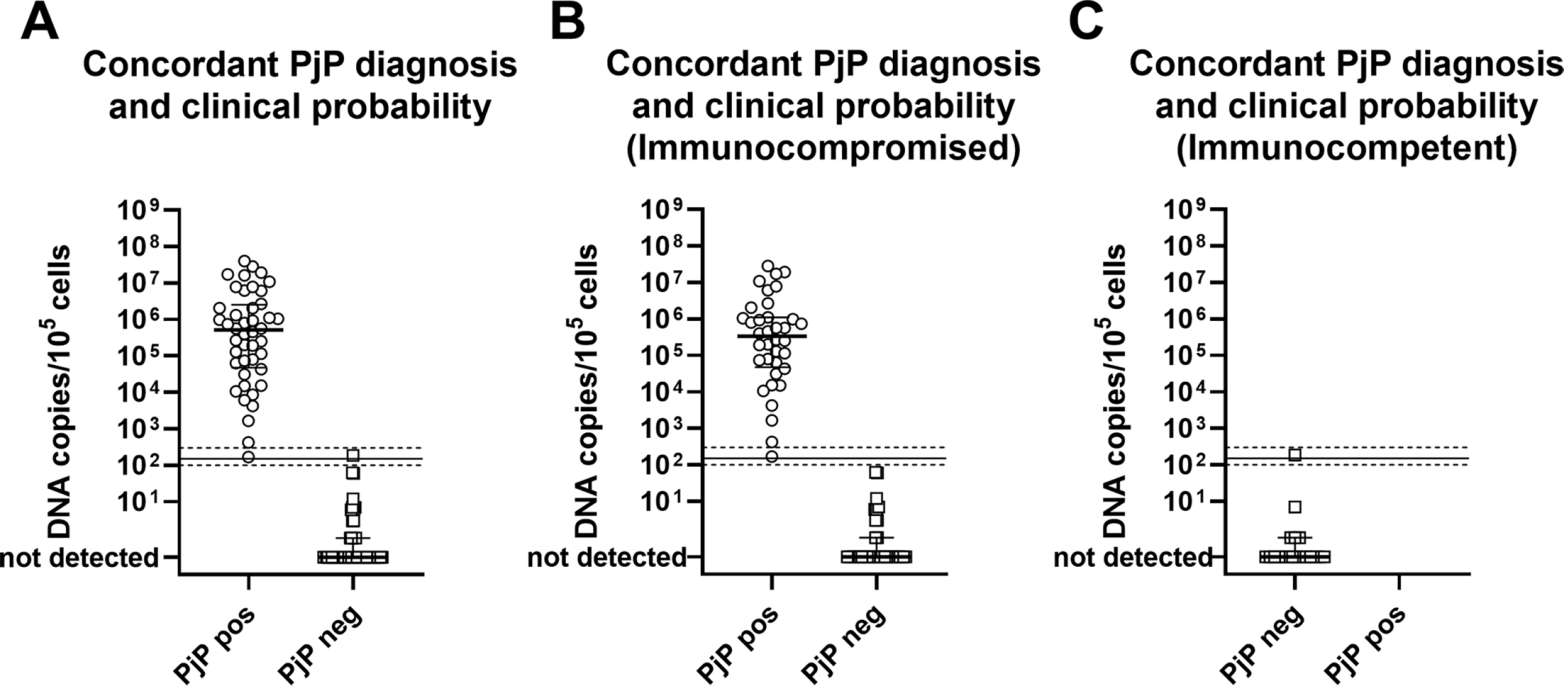
*P. jirovecii* DNA load in BAL or sputum of patients with positive (PjP pos) or negative (PjP neg) conventional diagnosis of PjP expressed per 10^5^ cells tested. Data are reported for patients with a positive or negative conventional diagnosis of PjP, associated with a high (HP) or low clinical probability (LP) of PjP (**A**), and for patients with a concordant conventional diagnosis and clinical suspicion of PjP, according to their immunocompromised (**B**) or immunocompetent (**C**) status. Horizontal solid line represents the optimal cut-off value (150 copies/10^5^ cells) for diagnosis of PjP, whereas horizontal dashed lines represent boundaries of a borderline zone (100–300 copies/10^5^ cells).

We repeated the analyses (Fig. 2A) considering only positive (44 patients) or negative (58 patients) patients for conventional detection of *P. jirovecii* in association with HP or LP of PjP, as previously discussed. We excluded one patient with positive detection of the microorganism associated with an LP of PjP, 32 patients with no detection of the microorganism but HP of PjP, and 28 patients without sufficient clinical data to perform a classification. The same cut-off could be applied, with an analytical sensitivity of 100% (95% CI: 92%–100%) and specificity of 98% (95% CI: 91%–100%). Results did not change significantly when we divided the patients with concordant detection of *P. jirovecii* and clinical suspicion according to their immunocompromise status (*n* = 75 immunocompromised individuals and *n* = 19 immunocompetent ones; Fig. 2B and C).

## DISCUSSION

The diagnosis of PjP is based on ME, but recently new molecular approaches have been developed to improve diagnosis in clinical settings (16, 24, 25). In our study, a total of 63 (38.6%) ME-positive samples were confirmed by using qPCR, thus suggesting a good sensitivity of the qPCR assay. Moreover, the detection of 21 additional positive samples by qPCR assay but negative with ME suggests a possible *P. jirovecii* colonization. In fact, 95.2% of them had a *P. jirovecii* load <100 DNA copies/10^5^ cells. The higher analytical sensitivity of molecular assays leads to an important consideration, suggesting that the qPCR assay is more sensitive to detect low fungal loads, but it may not always be able to distinguish clinically significant infections from colonization. Several studies have investigated a possible cut-off value of fungal load determined by qPCR to distinguish infection from colonization. The cut-off value obtained from data analysis on samples included in our study was 4,000 copies/mL. Notably, it is very similar to the ones reported in two other studies, ranging from 10^2.8^ copies/mL to 5 × 10^3^ copies/mL (25, 26). The variability of cut-off values may rely on the type of clinical specimens collected, their adequacy in terms of cellular concentration, and the assay’s intrinsic characteristics, such as the gene target or analytical sensitivity. Due to all these parameters, the evaluation of clinical performance of molecular assays is very difficult, and some authors suggested that it should be made by each individual institution (27, 28).

Since there is no standardized procedure to establish an appropriate cut-off value, we normalized the obtained results (already expressed in copies/mL) on the cellular DNA content obtained by β2-microglobulin quantification, and the results were then expressed in fungal DNA copies/10^5^ cells. After normalization, the cut-off value changed to 150 copies/10^5^ cells. To our knowledge, this is the first attempt to normalize the *P. jirovecii* DNA copies on cellular DNA. Moreover, since *P. jirovecii* is strictly associated with lung epithelium cells, the normalization of the number of cells collected in the samples allows a more standardized expression of the fungal load and, in addition, it may help in the definition of high-risk or low-risk cases in those patients with non-optimal samples.

From the data reported, all patients with fungal load <100 DNA copies/10^5^ cells were not affected by PjP, whereas patients with >300 DNA copies/10^5^ cells had confirmed PjP diagnosis. For those patients with fungal load between 100 and 300 DNA copies/10^5^ cells (borderline zone), it was not possible to distinguish between colonization and infection only using the qPCR value. Clinical evaluation in patients included in the borderline zone is then suggested.

In our study, only two patients (2/163, 1.2%) were included in the borderline zone. Among them, one HIV-AIDS patient with radiological findings suggestive of interstitial pneumonia had ME-confirmed PjP and low fungal load, probably due to a first phase of the infection; the other one was immunocompetent, with a suspected oncological malignancy but not diagnosed with PjP, thus suggesting a possible colonization of the lung environment.

In the context of multiple combined strategies for *P. jirovecii* diagnosis, qPCR may be a useful diagnostic tool in high-risk clinical settings, when radiological signs and serological markers support the hypothesis of an active fungal infection. However, it is essential to consider the clinical context of the patient when interpreting PCR results. This molecular approach enhances the reliability of the diagnosis and, in particular, the use of the cut-off value may help avoid misdiagnoses in patients with possible colonization and not clinically relevant infections.

Although colonization is clinically irrelevant, it could still be considered for epidemiologic data (28).

Our study has several limitations. First of all, we normalized fungal loads based on the cellular β2-microglobulin quantification; however, cystic forms of *P. jirovecii* may not be associated with epithelial cells, potentially leading to an overestimation of *P. jirovecii* levels. We also acknowledge that our sample size is relatively limited, and we are planning to increase the number of enrolled patients in the near future. Moreover, to our knowledge, this normalization method has never been used in the context of *P. jirovecii* diagnosis. Since this is a monocentric study, collaboration with other institutions would be highly recommended and beneficial to better validate the method.

In conclusion, our data support the qPCR as a reliable alternative to ME for PjP diagnosis; in fact, compared with traditional methods, qPCR is more reproducible, less operator-dependent, and has a short turnaround time.

However, in clinical cases in which fungal load is in the borderline zone (100–300 copies/10^5^ cells), a multidisciplinary approach, including ME, is still required.

Further studies are needed to investigate the role of *P. jirovecii* colonization in immunocompromised patients compared to immunocompetent individuals. Epidemiological data on circulating strains, obtained by genome sequencing, would also be valuable. Moreover, the detection of *P. jirovecii* from nasal/oropharyngeal swabs could be an interesting application of qPCR to be evaluated, thus avoiding invasive, costly, and operator-dependent procedures that require specialized personnel and involve high time and cost burdens.

## SUPPLEMENTAL MATERIAL

Table S1 spectrum.01563-25-s0001.docx Clinical suspicion criteria for PjP.

ASM does not own the copyrights to Supplemental Material that may be linked to, or accessed through, an article. The authors have granted ASM a non-exclusive, world-wide license to publish the Supplemental Material files. Please contact the corresponding author directly for reuse.
