# Supplementary material for: Real-time polymerase chain reaction (PCR) for Pneumocystis jirovecii detection in lower respiratory tract samples
Source: Microbiol Spectr. 2025 Nov 6;13(12):e01563-25. doi: 10.1128/spectrum.01563-25 (PMC12671181; doi:10.1128/spectrum.01563-25)
Supplement: Table S1 — Clinical suspicion criteria for PjP. [file spectrum.01563-25-s0001.docx]

| **Criteria** | **Description** |
| --- | --- |
| - Anamnestical Criteria for Suspected Diagnosis | - History of recent neutropenia (< 0.5 x 10^9 neutrophils/L for more than 10 days), temporally correlated with the onset of symptoms |
| - Allogeneic hematopoietic stem cell transplantation | - Prolonged corticosteroid therapy (> 3 weeks) |
| - Therapy with other immunosuppressive agents (cyclosporine, TNF-α inhibitors, specific monoclonal antibodies, or nucleoside analogs) within the 90 days prior to symptom onset | - Severe congenital immunodeficiency |
| - Imaging Criteria | - Signs of interstitial lung   disease/multiple consolidations |
| - Clinical Criteria | - Symptomatic triad (cough, fever, pneumonia) |
| - Biochemical Criteria | - Elevated LDH (Lactate Dehydrogenase) |
| - Positive circulating B-D-glucan test | - Inflammatory markers typically low or minimally elevated |

**Table S1.** Clinical suspicion criteria for PjP
